# Sleep-Related Painful Erections Following Sexual Intercourse

**DOI:** 10.1007/s10508-017-1132-0

**Published:** 2017-12-08

**Authors:** Pieter C. Barnhoorn, Woet L. Gianotten, Mels F. van Driel

**Affiliations:** 1 Department of Public Health and Primary Care, Leiden University Medical Center, Hippocratespad 21, Zone V0-P, PO Box 9600, 2300 RC Leiden, The Netherlands; 2 Department of Rehabilitation Sexology, ‘De Trappenberg’ Centre for Physical Rehabilitation, Huizen, The Netherlands; 3 Department of Urology, University Medical Center Groningen, Groningen, The Netherlands

**Keywords:** Sleep-related painful erections, Sleep-related erections, Erectio nocturna dolorosa, Nocturnal penile tumescence, Rapid eye movement (REM) sleep, Sexual intercourse

## Abstract

Sleep-related painful erections (SRPE) is a rare disorder characterized by recurrent painful nocturnal erections during REM sleep in the absence of pain during daytime erections. Approximately 35 cases of SRPE have been reported in the literature, none of them associated with preceding sexual intercourse. We add the report of a 40-year-old patient with a 6-year history of SRPE which only, but always, occurred after sexual intercourse with ejaculation in the evening before. As a result, the frequency of intercourse diminished, causing relationship problems. A non-pharmacological solution was found in shifting the time of sexual intercourse. The patient refused any proposed pharmacological treatment, because of “not wanting to be a patient at his age.”

## Case Report

A 40-year-old man (Caucasian, M.Sc., working as a fund manager, not married, not religious) reported that he had been suffering from sleep-related painful erections (SRPE) for 6 years. He awoke several times a night with a painful erection. The erection and the pain could be relieved by physical exercise or cooling the penis for about 3 min. Remarkably, he reported that his SRPE only occurred after having sexual intercourse, including ejaculation, the evening before. He did not have SRPE when intercourse and ejaculation had taken place during other times of the day. The duration of the intercourse did not make any difference; if he ejaculated during intercourse before he went to sleep, SRPE would occur in any case. After masturbation, he experienced no problems at all.

Because of these SRPE, the patient experienced significant problems in his romantic relationships. His former partner felt dissatisfied with the diminished frequency of intercourse, and, partly due to this, their relationship ended. The patient still experienced SRPE in his current relationship, which began 3 years after his former one ended. Because of his SRPE, the patient decided to stop having intercourse for a number of weeks. This led to slight relationship problems and caused his partner to advise him to consult the general practitioner (GP). He asked his GP whether he could rule out medical abnormalities and whether he could recommend a non-pharmacological therapy to remedy his complaints. The patient underwent a biopsychosocial evaluation by his GP, ruling out medical conditions, drug abuse, psychological issues, and major relationship problems. Physical examination, a neurological examination, examination of the external genitalia, digital rectal examination, blood pressure and pulse also did not show any abnormalities. Because the patient wanted to be sure there were no other medical abnormalities, he requested a referral to a urologist. Subsequent visits to the urologist provided the following information: Doppler ultrasound of his penile vessels performed after a night without SRPE was normal; nocturnal penile tumescence registration (NPTR) with a RigiScan device (Dacomed Corporation, Minneapolis, MN, USA) showed five periods of SREs, the last one with a duration of about 90 min. After the urologist visits, the patient was referred back to his GP. The patient refused any proposed pharmacological treatment, because of “not wanting to be a patient at his age.” Because his SRPE only occurred after having sexual intercourse the previous evening, his GP suggested the patient should have intercourse at other times of the day. As a result, the patient no longer experienced SRPE and this effect remained during the follow-up period of 3.5 years.

Unfortunately, despite this result, his current relationship ended for other reasons than shifting the time of sexual intercourse. In the last 3 years, he has had some short-lived relationships. He now is a schooled practitioner of tantric sex (defined by him as practices in which slow, mindful, non-orgasmic sexual union, or masturbation creates a path to the experience of spiritual ecstasy) and aims to eliminate all orgasms, both during intercourse and masturbation. Only a few times, in the last 3.5 years, he had sexual intercourse, including ejaculation in the evening. Without exception, these were followed by awakenings several times a night with a painful erection.

## Discussion

Sleep-related erections have been known since antiquity (van Driel, 2014). However, only in the late 1930s did scientists start investigating SREs systematically. Ohlmeyer, Brilmayer, and Hüllstrung (1944) first identified that SREs take place in cycles. They described these erections occurring every night, approximately every 85 min, and lasting approximately 25 min. As rapid eye movement (REM) sleep follows the same cyclical pattern, Fisher, Gross, and Zuch (1965) hypothesized that these nocturnal erections occurred during REM sleep (Abouda, Jomni, Yangui, Charfi, & Arnulf, 2016; Aserinsky & Kleitman, 1953; Ferre et al., 2012).

SREs naturally and involuntarily occur in all healthy men. They first occur prenatally, and in that period they are also strongly associated with REM sleep (Koyanagi, Horimoto, & Nakano, 1991). SREs continue into old age, but during a man’s lifetime they decrease with regard to duration and intensity. SREs can therefore be considered as a function of age. There are far fewer studies on female nocturnal genital physiology, but women also experience REM sleep-related genital vascular engorgement (Fisher et al., 1983).

Unfortunately, some men experience painful SREs which disrupt their sleep pattern: the so-called SRPE (Abouda et al., 2016). SRPE were first described in 1939 by Walker and Strauss in their book called *Sexual Disorders in the Male* (Walker & Strauss, 1948). In the following years, little research was published on SRPE. Even now, the phenomenon of SRPE is not well understood. In the acute moment, the patient with SRPE wakes up because of deep pain inside the erect penis. This pain can be relieved by physical exercises (e.g., abduction and flexion of the hips and walking) or by cooling of the penis with ice cubes or a cold shower (van Driel, Beck, Elzevier, van der Hoeven, & Nijman, 2008). Many drugs have been used to prevent SRPE: anti-psychotics, anti-depressants, benzodiazepines, anti-epileptics, digoxine, beta2 agonists, anti-androgens, 5-alpha-reductase inhibitors, and baclofen (van Driel et al., 2008). They represent different psychopharmacological approaches: drug-induced blocking of erections, suppressing REM sleep, or suppressing pain and promoting relaxation of pelvic floor muscles including the bulbospongiosus and ischiocavernosus. Weighing the advantages and disadvantages, baclofen seems the best initial pharmacological treatment option (van Driel et al., 2008). However, not every patient with SRPE is open to a pharmacological intervention. In the case of SRPE which only occur after sexual intercourse during the previous evening, changing the time of sex appears to be a simple non-pharmacological solution.

It is tempting to speculate about what is behind the SRPE in this patient. We maybe have some explanation. It could be that the deflating mechanism in this patient is deficient. An indication for that is the very long (90’) duration of sleep-related erection found at nocturnal registration. Over the past decades, much knowledge has been developed on preventing deflating. In that process, the phosphodiesterase-5 inhibitors (erection pills) have been discovered. This group of patients with SRPE maybe needs an approach in the opposite direction. On the other hand, we do not have an explanation why the phenomenon in this patient only took place after having had intercourse before the night.
